# Association of European Border Regions (AEBR)

**DOI:** 10.1093/eurpub/ckac129.488

**Published:** 2022-10-25

**Authors:** M Guillermo Ramirez

**Affiliations:** Association of European Border Regions, Berlin, Germany

## Abstract

AEBR is one of the oldest regional associations in Europe (founded in 1971), with a hundred members (border and cross-border regions) in more than thirty European countries. AEBR works for the interest of border regions towards EU and national authorities, developing capacities, increasing awareness, organizing events and implementing projects. AEBR currently implements IVY (Interreg Volunteer Youth) on behalf of the European Commission's DG Regio, within the framework of the European Solidarity Corps, which has deployed more than 700 young Europeans in Interreg programmes and projects during the last four years. It also manages b-solutions to tackle cross-border legal and administrative obstacles and test possible solutions, and takes part in other projects in Europe and other continents. It has recently finished a study for DG SANTE on cross-border patients’ flows in various EU cross-border areas. The association will be presented by it's Secretary General, Martin Guillermo-Ramírez.

